# The 2024 French guidelines for scenario design in simulation-based education: manikin-based immersive simulation, simulated participant-based immersive simulation and procedural simulation

**DOI:** 10.1080/10872981.2024.2363006

**Published:** 2024-06-06

**Authors:** Guillaume Der Sahakian, Maxime de Varenne, Clément Buléon, Guillaume Alinier, Christian Balmer, Antonia Blanié, Bertrand Bech, Anne Bellot, Hamdi Boubaker, Nadège Dubois, Francisco Guevara, Erwan Guillouet, Jean-Claude Granry, Morgan Jaffrelot, François Lecomte, Fernande Lois, Mohammed Mouhaoui, Ollivier Ortolé, Méryl Paquay, Justine Piazza, Marie Pittaco, Patrick Plaisance, Dan Benhamou, Gilles Chiniara, Etienne Rivière

**Affiliations:** a Department of Emergency Medicine, Centre Hospitalier d’Orange, Orange, France; b Department of Anesthesiology and Intensive Care, Université Laval, Québec, QC, Canada; c Center for Medical Simulation, Liège University Hospital, Liège Belgium; d Center for Medical Simulation, Boston, MA, USA; e Department of Anesthesiology, Intensive Care and Perioperative Medicine, Caen Normandy University Hospital, Caen, France; f School of Health and Social Work, University of Hertfordshire, Hatfield, Hertfordshire, UK; g Faculty of Health and Life Sciences, Northumbria University, Newcastle Upon Tyne, UK; h Hamad Medical Corporation Ambulance Service, Doha, Qatar; i Paediatric Cardiology, Paediatric Heart Center, Department of Surgery, University Children’s Hospital, Zurich, Switzerland; j Department of Anesthesiology, Intensive Care and Perioperative Medicine, Kremlin Bicêtre University Hospital, APHP, Paris, France; k Centre de simulation NorSimS & Service de néonatalogie, Centre Hospitalier Universitaire de Caen Normandie & Université de Caen-Normandie, Caen, France; l Emergency Department, Fattouma Bourguiba University Hospital & Research Laboratory, University of Monastir, Monastir, Tunisia; m Department of Emergency Medicine, Quartier Hôpital, University Hospital of Liege & Center for Medical Simulation, Liège University Hospital, Liège, Belgium; n Chargé de projets en simulation continue et initiale, Cadre de santé formateur en simulation en santé, IFSI Croix Saint Simon, Montreuil, France; o AllSims Center for Simulation in Healthcare, University Hospital of Angers, Angers, France; p Independent Consultant in Simulation, Brest, France; q Department of Emergency Medicine, Cochin University Hospital, APHP, Paris, France; r Department of Anesthesiology, Intensive Care and Perioperative Medicine, Liège University Hospital, Liège, Belgium; s Faculty of Medicine and Pharmacy, Hassan II University, Casablanca, Morocco; t Department of Emergency Medicine & CESU Martinique, University Hospital Center of Martinique, Fort-de-France, France; u Department of Emergency Medicine, Lariboisière University Hospital, APHP, Université de Paris & ILumens Platform of Medical Simulation Paris University, Paris, France; v Internal Medicine and Infectious Diseases unit, Haut-Leveque Hospital, University Hospital Centre of Bordeaux, Pessac Cedex, France; w Faculty of Medicine, Bordeaux University, Bordeaux, France

**Keywords:** Simulation-based education, scenario design, simulated patient, procedural simulation, immersive simulation, crisis resource management

## Abstract

**Background:**

Simulation-based education in healthcare encompasses a wide array of modalities aimed at providing realistic clinical experiences supported by meticulously designed scenarios. The French-speaking Society for Simulation in Healthcare (SoFraSimS) has developed guidelines to assist educators in the design of scenarios for manikin- or simulated participant- based immersive simulation and procedural simulation, the three mainly used modalities.

**Methods:**

After establishing a French-speaking group of experts within the SoFraSimS network, we performed an extensive literature review with theory-informed practices and personal experiences. We used this approach identify the essential criteria for practice-based scenario design within the three simulation modalities.

**Results:**

We present three comprehensive templates for creating innovative scenarios and simulation sessions, each tailored to the specific characteristics of a simulation modality. The SoFraSimS templates include five sections distributed between the three modalities. The first section contextualizes the scenario by describing the practicalities of the setting, the instructors and learners, and its connection to the educational program. The second section outlines the learning objectives. The third lists all the elements necessary during the preparation phase, describing the educational method used for procedural simulation (such as demonstration, discovery, mastery learning, and deliberate practice). The fourth section addresses the simulation phase, detailing the behaviors the instructor aims to analyze, the embedded triggers, and the anticipated impact on simulation proceedings (natural feedback). This ensures maximum control over the learning experience. Finally, the fifth section compiles elements for post-simulation modifications to enhance future iterations.

**Conclusion:**

We trust that these guidelines will prove valuable to educators seeking to implement simulation-based education and contribute to the standardization of scenarios for healthcare students and professionals. This standardization aims to facilitate communication, comparison of practices and collaboration across different learning and healthcare institutions.

## Summary

‘What this article adds’

1. The SoFraSimS provides guidelines to facilitate the development of simulation-based activities.

2. These guidelines are theory-informed as well as evidence and experience-based.

3. A detailed approach to writing a complete activity or scenario for procedural and immersive simulation including manikins or simulated participants is provided (the ‘SoFraSimS templates’).

4. This work aims at standardizing practices and exchanging scenarios between simulation centers.

## Introduction

Healthcare simulation is now widely employed for training healthcare professionals and students, with an ever-growing body of evidence supporting its efficacy as a learning strategy [1,2]. Ensuring the optimal use of simulation tools for learning necessitates meticulous scenario design to guarantee an authentic learning experience for participants [3], while also ensuring that learning objectives are appropriately formulated and achieved. This intricate process of developing a simulation-based learning experience is herein referred to as ‘activity design’, ‘script design’, or ‘scenario design.’ The complexity of this procedure can be streamlined through the application of instructional design principles, particularly the A.D.D.I.E. model [4].

Earlier work, specifically the 2015 publication of the TEACH Sim tool for systematic scenario design, introduced crucial components for inclusion in a scenario template, emphasizing accessibility to educators, applicability to specific simulators or disciplines, comprehensive participant information, instructional methodologies, design strategies, and support details [5]. Subsequent publications have predominantly focused on scenario conception [6–8]. Additionally, there has been a proliferation of freely accessible online templates; however, there is a conspicuous absence of templates tailored for procedural simulation, lacking both expert-driven and evidence-based consensus on activity or scenario content. Indeed, to the best of our knowledge, there is no scenario template officially proposed by international simulation societies for procedural and immersive simulation, involving either manikins or simulated participants.

Consequently, in 2022, the French-speaking Society for Simulation in Healthcare (SoFraSimS) established a task force to develop three reference templates aimed at assisting educators in designing activities and scenarios for both procedural, manikin- and simulated participant- based immersive simulation, and intended for unrestricted dissemination. We succinctly presented in this article the findings derived from this year-long endeavor.

## Methods

### Literature search strategy

To acquire a broad vision of how to write a scenario, we performed a literature search on MEDLINE, Web of Science, PubMed Central, ERIC, and Google Scholar. Search terms included ‘Healthcare simulation template’, ‘Simulation template’, ‘Simulation scenario’, ‘Simulation scenario design’, ‘Simulation scenario conception’, ‘standardized simulation template’ and ‘standardized simulation scenario’. We also based our work on freely available templates used for scenario design that can be found on the internet, among them the templates from California Simulation Alliance (CSA) [9], Sim Tech [10], Emergency (EM) Sim Cases [11], the Alfred Intensive Care Unit [12], the UCI simulation center [13], the medical college of Wisconsin [14], the HETI guide [15], the national league for nursing [16], The American Council of Academic Physical Therapy (ACAPT) [17], Stanford University [18], Frimley health NHS foundation trust [19], NHS Wales [20], the Office of inter-professional education of Alabama [21], and Simulation Canada [22]. All these documents served as a baseline study to select the essential criteria for scenario design.

### Setting up the SoFraSimS 2023 French-speaking working group of experts (2023-SFWGE)

The 2023-SFWGE was intended to be composed of a large (25 members, *see supplemental Table S1*), French-speaking (France, Belgium, Canada, Switzerland, Morocco, Tunisia) and inter-professional (medical doctors, nurses, educators, and simulation technicians) cohort of simulation experts. The call for application was made through the SoFraSimS network. The 2023-SFWGE has expertise in both Simulation-Based Education (SBE) and healthcare, and includes a diverse mix of clinical and academic backgrounds. All its members currently work or have previously worked in a hospital or an academic clinical simulation center for at least ten years. Many also have international experience within the board of directors or Executive Committee of the SoFraSimS, the International Nursing Association for Clinical Simulation and Learning (INACSL), the UK Association for Simulated Practice in Healthcare (ASPiH), the Society in Europe for Simulation Applied to Medicine (SESAM), the Society for Simulation in Healthcare (SSIH), and the precursor to Simulation Canada. Over the last 15 years, most have published works or participated in the elaboration of national guidelines on the use of SBE in healthcare. Finally, all regularly write scenarios for pre- and post-graduate training in healthcare, and for inter-professional and multi-institutional (educational and clinical) simulation-based activities.

### Developing the activity and scenario templates

Following the exhaustive literature review, we structured the development of the templates in three steps. The first consisted of specific focus groups within SFWGE to determine quality criteria for an optimal activity and scenario in procedural, manikin- and simulated participant- based immersive simulation The second was to design three blank scenario templates in French, in the focus groups optimized the blank scenario templates during the SoFraSimS symposium in Nice (France) in 2023. The final step was to validate these templates with the help of the nominal group method [23,24], a structured method of exchanges (e-mail, focus groups) between experts to reach a consensus. The two main authors of this article (GDS & ER) led the nominal group.

## Results

SBE is an educational strategy that requires a large amount of material, human, and financial resources [25,26]. Therefore, its use must be carefully weighted as previously described [27], and the educator in charge of a simulation session must do whatever is possible to keep control over the simulation activity and reach the targeted learning objectives [28,29].

### Results of the first step: literature search

As a starting point, according to previously published works [5,30,31], a scenario template requires eight items including a title, learners’ description with the target group size as well as their level of expertise and any other prerequisites, level of difficulty adapted to the learners, clearly defined and targeted learning objectives devised, whenever possible, according to the SMART method [32,33], material and human resources, technical aspects of the simulator (settings, physiological parameters etc.), description of the environment, handouts to be provided during the scenario (ECG, X-ray, prescriptions, etc.), bibliography and notes (elements for improving the scenario, debriefing aids, etc.).

In 2008, Dieckmann & Rall proposed the Tupass scenario script [34], which is a fairly complete 7-page scenario model combining a summary of the scenario, the learning objectives and major debriefing points, briefing, human and material resources, and the various roles to be assigned (simulated participants). The Tupass scenario script contains a section dedicated to ‘scenario life savers’, which are techniques or methods for rescuing the scenario if the learners deviate too much from the learning objectives [35].

Waxman (2010) [36], and Bambini (2016) [6] published a scenario writing reference framework for nursing educators, which included the level of complexity and the level of fidelity.

In 2012, in France, Granry and Moll proposed guidelines for the adequate use of SBE in healthcare [37], in which they stipulated that each scenario should be written according to a formalized plan that includes technical (medical expertise) and non-technical (Crisis Resource Management (CRM) and soft skills) learning objectives, the sequence of the training session (duration, instructor/learner ratio, simulation sequence), the main debriefing points, the methods for assessing learners and a bibliography.

In 2018, Jaffrelot et al. [38] suggested five essential elements for writing a scenario: (1) identification of the learning objectives consistent with the competencies to be acquired by the learners; (2) previous knowledge/competencies of the learners on which to base learning (prerequisites, profession, professional issues); (3) previous knowledge of the curriculum to better integrate the simulation session into the learners’ training program; (4) identification of material and human resources; and (5) an authentic medical case for adequate contextualization of the learning process.

Finally, in 2019, Der Sahakian et al. [39] proposed 6 essential criteria for writing a scenario: (1) the writing should be directed by an educator trained in simulation, (2) the scenario should be based on ‘valid’ learning objectives, (3) it should be inspired by real-life cases and include the progressive sequence of events, (4) its level of complexity should be adapted to the learners, (5) the material and human resources must be anticipated, and (6) it should include a briefing as often as possible.

Consequently, the literature review and the various published templates highlighted 6 parts required to adequately describe a scenario: (1) a complete layout with a concise title, (2) practical elements (author, revision date, supporting documents, bibliography, etc.), (3) a well-identified learner population and adequate knowledge regarding their curriculum or simulation exposure, (4) clear, limited, descriptive, easily observable and measurable learning objectives to develop technical and non-technical competencies, (5) all material and human resources necessary for an authentic case (adequate simulator and environment, medical file, scenario life-savers, etc.), (6) a detailed timeline of the session (concise briefing, sequence of events during the activity or scenario, main debriefing points linked to the learning objectives), including a section in which improvements are suggested to strengthen future sessions. We also believe that, in the last section, educators designing scenarios must attest to the degree of control they have over the simulated experience (contrary to a real-life situation) to ensure the learners’ psychological safety [40] and attainment of the learning objectives, by recording how observable behaviors by learners can be triggered and how they will impact the course of the simulation [41]. This is further explained below.

### Results of the second and third steps: template design and validation

We aimed at introducing simulation templates for the three most frequently used simulation modalities, i.e., procedural, manikin- or simulated participant- based immersive simulation

*1-Manikin-based immersive simulation scenario (appendix 1)*

1. **Scenario context**, with practical information specific to the scenario: a title (that does not divulge the case), the simulation institution, the author’s name with contact information, a review date or version number (when modifications are done after specific feedback), the educational team names and contact information, a summary of the case for the instructors, the target learner population (number, profession, previous experience with SBE), and the link, if any, to an educational program.
2. **Learning objectives** in response to the educational specific needs, limited to 3 to 5, written as SMART objectives whenever possible, including both technical and non-technical objectives; the section also includes the interdisciplinary and/or inter-professional nature of the team, whether the activity is aimed at pre- or post-graduate learning, and potential emerging learning goals, either theoretically anticipated or documented after the scenario has been run a few times.
3. **Scenario preparation**, which lists all technical elements for the scenario: learner handouts during the simulation, educational document(s) or summaries to be given to learners before and/or after the session, bibliographical references or guideline(s) on which the scenario is based, specific information to be given to facilitators or other participants during the simulation (‘scenario life-savers’), and a detailed description of the environment and required equipment^42^.
4. **The course of the simulation**, including potential elements specific to the case to be included in the learners’ prebriefing^43^, a briefing that must be concise, the duration of the scenario. Importantly, this section must include the successive stages of the scenario that enable the observation of *learners’ behaviors* that demonstrate the achievement (or lack thereof) of specific learning objectives. It must also describe the *triggering events* (‘triggers’) embedded within the scenario, which allow the emergence of the observable behaviors. Finally, it must describe the expected impact these observable behaviors will have on the future course of the simulation (*natural feedback*, Figure 1). These three crucial elements aim to guarantee maximum control over the simulation experience to ensure the learners’ psychological safety and achievement of the learning objectives (for more detailed information about this part, see ref^41^). This section also includes anticipated learning points and strategies to be used during the debriefing^44,45^ based on learning outcomes.

**Figure 1.**
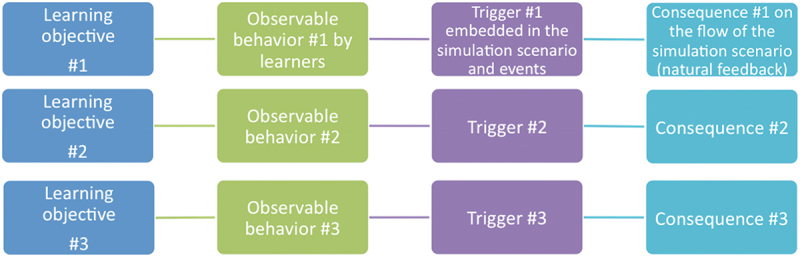
How learning objectives should be linked to observable behaviors and adequately triggered with natural feedback when writing an immersive scenario. (adapted from [41].
5. **Scenario quality control**, including the history of scenario piloting (or ‘dry runs’) and any element that would iteratively improve the scenario upon each occurrence until an adequate and stable state is reached.

*2-Simulated Participant (SP) based immersive simulation scenario (appendix 2)*

We chose to use the term ‘simulated participant (SP)’ rather than ‘simulated’ or ‘standardized patient’, following the recommendations of the Association of Standardized Patient Educators published in 2017: ‘The term “simulated participant” is being used as a more inclusive term to refer to all human role played in any simulation context’ [46]. The SP encompasses not only the patient, but also a family member or any other person directly involved in the scenario. The SP scenario template is based on that for manikin-based immersive simulation, but focuses on care relationships, and may involve patients and/or family members, or colleagues. It has the same five scenario sections, with some specific features regarding the course of the simulation session, adding three sub-sections:

- **The briefing and acting role** of the SP: reason for consultation, sex, age, identity, medical history and background, treatments and allergies, family, professional activity, leisure activities, last meal, height/weight, symptoms, usual behavior and speech patterns, emotional state, physical signs to be simulated, timing of the delivery of additional information to the learners;
- **SP presentation**: clothes, makeup, initial state and posture, environment, and room setup;
- **The starting sentence and all the various stages** with the learners’ observable behaviors (related to the learning objectives) and how these are triggered with natural feedback, as explained above.

*Procedural simulation scenario (appendix 3)*

Of note, no template for writing an activity or scenario for procedural tasks currently exists in the literature. This scenario concerns the development of psychomotor skills in healthcare and their associated procedures, i.e., bedside examination and surgical procedures. As a preamble, we recommend the following instructional design steps [47]

1. Clearly define the learning objectives for each procedure;
2. Carefully choose the simulation modality: part-task trainer only or hybrid simulation with a simulated participant;
3. Vary the characteristics of the learning tasks as much as possible, and consider using a mastery learning approach (learning in steps of increasing difficulty until mastery is achieved, with an assessment after each step);
4. Provide a short clinical vignette to put the simulation activity in its appropriate context (for example, a case of bacterial meningitis requiring a lumbar puncture with no contraindication, evidencing a bacteria in the cerebrospinal fluid and triggering the discussion for further antimicrobial agents and follow-up);
5. Provide feedback on the entire procedure (process feedback) rather than solely on the individual movements or the overall outcome (success/failure);
6. Use validated or in-house assessment scales to assess learning at each stage;
7. Anticipate strategies to prevent competency decay: encourage self-regulated learning and deliberate practice^48^ (autonomous practice with free access to the simulator once learning is achieved, with specific feedback and targeted objectives), schedule skill maintenance sessions every 6 to 12 months, and use mental imagery practice^49^ before or after sessions to mentally rehearse the procedure.

The procedural activity or scenario template has 5 parts:

1. **The scenario context;**
2. **The learning objectives and planned learning strategies**: discovery-based learning (active experimentation with problem-solving), demonstration or exemplification (observation and imitation of procedures), mastery learning and/or deliberate practice;
3. **The preparation**: associated supporting documents and handouts (including videos and mental imagery technique), bibliographical references and guidelines on which the scenario is based, authentic environment and equipment (immediately available or on demand), and the number of required task-trainers or simulators taking into account the best simulator-to-learner ratio possible^50^;
4. **The ‘simulation’ process**: pre-briefing including some elements specific to the procedure with role assignment, initial presentation of the simulator(s) and on-demand clinical/biological/radiological handouts whenever appropriate, detailed sequence of activities, briefing to introduce the clinical case and define roles, the assessment scales (ideally evidence-based, or in-house if none exists), and the type of required feedback (simultaneous or delayed, individual or group, peer or instructor-oriented, self-regulated);
5. **Scenario quality**: list of items to improve the scenario to be determined after each simulation session, systematic simulator quality control, and strategies for preventing competency decay.

## Discussion

We described herein three templates for writing scenarios for procedural, and manikin- or simulated participant- based immersive simulation designed, and agreed upon by SoFraSimS. We based the SoFraSimS templates on the existing literature, published frameworks, and the long-term experience of experts in the field, through a strategy of nominal groups.

To date, Benishek’s TeachSim [5] using the SMARTER methodology is one of the most accomplished works on the subject especially for immersive simulation. To explain how our guidelines and templates improve upon similar templates, and how they can result in more effective simulation-based education, we first compared them to the TEACH template (Table 1). Many educational and practical factors were added, especially for immersive simulation. Practical factors such as the location of the scenario, the authors’ contact information, and the number of targeted learners provided needed information for the learners and the simulation team. The link between the simulation scenario and an educational program is required in order to use simulation as a complementary educational tool to other learning media and activities. To that end, we asked the scenario authors to specify the needs assessment on which the scenario was grounded to ensure it answers specific learning needs. Learning objectives were limited and divided into technical and non-technical, not merely to list learning objectives, but to make sure at least some human factor objectives (or crisis resource management competencies) are targeted in critical situations [51]. We anticipated plausible emerging learning objectives as those can be hugely unsettling for simulation instructors when they occur, therefore providing a minimal set of strategies not directly linked to the learning objectives if, unfortunately, learners deviate from them. We added a section to anticipate potential cognitive aids for learners as they have shown to be crucial and positively impact the quality and safety of care [52–54]. Specific elements presented to learners during pre-briefing and briefing are pivotal to reduce learner anxiety and adequately prepare them for this learning activity [55]. Regarding the timing, we added simulation duration and a dedicated time for learners to prepare before the clinical case begins for psychological safety, emotion control and mindfulness [56].

**Table 1.** Comparison between the TEACH and the SoFraSimS Templates.

		TEACH template	SoFraSimS templates
SCENARIO CONTEXT	Year	2015	2023
	Language	English (USA)	English (translated from French)
	Simulation modality	Immersive simulation	Procedural and immersive simulation including simulated participants
	Type of simulator	Manikin and human	Manikin and human
	Discipline	Any HCP	Any HCP
	Describes learning location	No	Yes
	Provides contact to reach the author	No	Yes
	Requires a case summary	Yes	Yes
	Describes purpose before context	Yes	Yes
	Targeted population of learners	Yes	Yes
	Specifies the number of learners	No	Yes
	Details participation prerequisites	Yes	Yes
	Links the scenario to a specific educational program	No	Yes
	Distinguishes scenario cast members	Yes	Yes
TRAINING OBJECTIVES	Requires adaptation to the results of learning needs assessment	No	Yes
	Identifies learning objectives	Yes	Yes
	Divides learning objectives into technical and non-technical knowledge, skills and attitudes	No	Yes
	Explicitly links learning objectives to content	Yes	Yes
	Limits learning objectives	Yes (<5)	Yes (<5)
	Anticipates plausible emergent learning objectives	No	Yes
SCENARIO PREPARATION	Requires references for the scenario	Yes	Yes
	Describes associated didactics	No	Yes
	Identifies supporting documents	No	Yes
	Anticipates potentially useful cognitive aids	No	Yes
	Provides equipment or prop options	Yes	Yes
	Labels type of simulator needed	Yes	Yes
	Depicts patient data	Yes	Yes
	Details simulator and equipment setup	No	Yes
	Anticipates specific elements to be presented to learners during pre-briefing?	No	Yes
SIMULATION PROCESS	Scenario briefing to learners	No	Yes
	Simulation duration	No	Yes
	Dedicated time for learners to prepare	No	Yes
	Initial status of the simulator	No	Yes
	Triggering events	Yes	Yes
	Identifies expected participant actions (observable behaviors)	Yes	Yes
	Scripts cues to transition between events (‘natural feedback’)	No	Yes
	Method of ending/exiting the scenario	No	Yes
	Prepares debrief or guided study plan	No	Yes
QUALITY IMPROVEMENT	Documents pilot testing	No	Yes
	Elements to improve the scenario for its further occurrence	No	Yes

The ‘natural feedback’ section provides specific cues to transition between the events of the scenario, thus reinforcing its fluidity. This aims to systematically ‘force’ the scenario authors to maintain control over the simulated experience to reach the set learning objectives. As such, we highlighted a new method to formalize learning objectives as observable behaviors that specific events trigger, with an anticipated impact on the simulated situation (natural feedback).

We further added the initial status of the simulator for manikin-based immersive simulations to ensure its readiness, and methods of ending a scenario once learning objectives are achieved or a specific event is reached. Specific points to be addressed during debriefing such as its duration, the strategies to solve the clinical problem and recontextualize are anticipated to foster personalized learning and foster transfer [44]. Finally, elements for quality improvement of the scenario are mandatory to improve it iteratively over successive simulation sessions. All the elements of the SoFraSimS templates discussed above were not included in the previous published templates.

Furthermore, we wanted to extend the landscape to the three most frequently used simulation modalities in healthcare education, design a template for patient-based immersive simulation in lines with the guidelines from the association of standardized patient educators (ASPE) [46], and provide a template for procedural simulation that did not exist to date.

Our work has some limitations and strengths. It partially relies on expert opinion and is therefore not entirely evidence-based. It also requires a substantial amount of time and rigor for educators to design a scenario. However, we believe that these structured templates will help educators who have or will adopt SBE to rigorously write adequate and complete scenarios to reach targeted learning objectives. These templates might help to standardize comparison and impact research in SBE by providing common models of scenarios. We also believe these templates might be useful to build a cooperative database of reviewed, and validated scenarios sharable across institutions. Of course, these templates aimed at collecting the most valuable information for scenario writing and design and are not meant as constraints on the creativity of any educator who might want to look for novel uses or innovations within SBE, especially in research projects.

## Conclusion

We presented herein the French guidelines and templates for the design of scenarios for procedural, manikin- and simulated participant- based immersive simulation. These three ready-to-use templates should be used as a solid foundation for creating state-of-the-art simulation sessions.

## Supplementary Material

Supplemental Material

## Data Availability

All data can be available upon request to the corresponding author.
