# Supplementary material for: The 2024 French guidelines for scenario design in simulation-based education: manikin-based immersive simulation, simulated participant-based immersive simulation and procedural simulation
Source: Med Educ Online. 2024 Jun 6;29(1):2363006. doi: 10.1080/10872981.2024.2363006 (PMC11164058; doi:10.1080/10872981.2024.2363006)
Supplement: Supplemental Material [file ZMEO_A_2363006_SM0364.docx]

**APPENDIX 1. Manikin-based immersive simulation scenario (translated from French)**

| *Institutional logo*  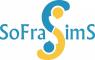  **MANIKIN-BASED IMMERSIVE SIMULATION**  **SCENARIO**  ***Scenario title (specify)*** | | | | | |
| --- | --- | --- | --- | --- | --- |
| *Manikin-based Immersive simulation refers to a scenario where learners are immersed in an environment close to reality with interactions among themselves, with the patient (a full-body patient simulator), with the environment, or with other participants in the simulation session (actors).* | | | | | |
| **SCENARIO CONTEXT** | | | | | |
| Simulation institution | *Name of the training institution* | | | | |
| Author(s) | *Person(s) who designed the scenario* | | | | |
| E-mail | *Contact person's e-mail for the scenario* | | | | |
| Revision date | *Date of last scenario revision* | | | | |
| Educational team (specify if trained in simulation) | *Technicians, facilitators, instructors, simulator voices...* | | | | |
| Brief summary for TRAINERS | *Scenario description exclusively for use by the educational team* | | | | |
| Target learners | Number | Function (profession, attire) | | | Prior experience with simulation |
|  |  |  | | |  |
| Possible link to a training program or curriculum | *Where does the scenario fit into the learners' training program? Professional development, initial training with a formal curriculum, postgraduate training...* | | | | |
| **TRAINING OBJECTIVES** | | | | | |
| Specific educational needs of the learners | | | | | |
| *Specify the method and results of the needs analysis that led to this scenario: “What justifies this scenario?”* | | | | | |
| **Learning objectives (3 to 5 max)** | | | | | |
| Technical skills | | | Non-technical skills | | |
| *Healthcare expertise adapted to the learners*  *Be able to ...* | | | *Human factors adapted to the learners*  *Be able to....* | | |
| Possible emerging learning objectives | | | | | |
| *Specify here any potential emerging objective that could be debriefed if necessary (to help prepare for debriefing).* | | | | | |
| **SCENARIO PREPARATION** | | | | | |
| Provided handouts during simulation, if necessary | *Documents, patient chart, bio, ECG, imaging...* | | | | |
| Learning aids to be provided to learners before and/or after the training session | *Cognitive aids or documentation from the available literature or developed by the educational team* | | | | |
| Bibliographical references or recommendations related to the scenario | *Specify here, if available, the bibliographical references on which the scenario is based* | | | | |
| Specific information for additional speaking roles during the simulation session | *Roles of stakeholders (facilitators, stressors, etc.), specific phrases, attitudes, etc.* | | | | |
| Choice of the simulation environment | *In situ, emergency room, hospitalization room, intensive care unit, etc.* | | | | |
| Description of the equipment required in the simulation room with a level of realism adapted to the learners’ expertise | *Description of the simulator (and actors/facilitators, etc.) including any makeup and consumables.* | | | | |
| Prebriefing at the start of the training session | *Invitation modalities, prebriefing adapted to the learners' simulation experience, specific information about the training session in which the scenario is integrated (e.g. death in simulation, etc.), prebriefing checklist used (to adequately prepare learners and avoid difficult debriefings).* | | | | |
| **SIMULATION PROCESS** | | | | | |
| Briefing for learners | *Individual or group*  *Case presentation focusing on relevant details: medical and surgical history, habitus, medication, morphological parameters, etc.* | | | | |
| Simulation duration | *Approximate duration of the simulation session, especially to fit the schedule of the day if necessary.* | | | | |
| *The following section will help you identify all the behaviors you want to observe in your learners concerning the scenario objectives. It is then necessary to identify all the triggers for these behaviors and anticipate their consequences or impact on the evolution of the situation.*  ***Insert or delete as many lines as you need, based on your objectives*** | | | | | |
| Preparatory phase for learners  (“immersion chamber”) | *Dedicated concentration time for learners before starting the simulation, preferably outside the simulation room and in a quiet environment* | | | | |
| Status / Duration (D) /  Trigger Event (TE) | **Observable learner behavior** | | | **Consequences on the situation (patient, environment, actor entry/exit, etc.)** | |
| *Basic clinical status of the simulator (hemodynamic parameters, starting sentence, posture, etc.)* | | | | | |
| State 1: *example: Initial care*  D: *5 minutes*  TE: *Arrival of the medical team* | *Team handover*  *Conditioning* | | | *Anticipated responses*  *The reaction of the patient/actors/environment to the learners' behavior* | |
| State 2:  D:  TE: |  | | |  | |
| State 3:  D:  TE: |  | | |  | |
| Condition 4:  D:  TE: | *Provide as many TEs or states as necessary for the scenario* | | |  | |
| *Method of ending and exiting the scenario* | | | | | |
| Preparing for debriefing | *1- Approximate planned debriefing duration*  *2- Strategies for resolving the simulated situation (chain of survival, ACR, SAED, ethical principles, etc.), emerging problems, etc.*  *3- Prepare strategies for transfer of learning to practice = plan some elements of recontextualization, i.e. show that the strategies used in the simulation are valid for other situations (including real-life situations), sometimes relying on the learners’ lived experiences if any, by analyzing with them what was identical and what was different from the simulated situation...* | | | | |
| **QUALITY** | | | | | |
| **History of scenario piloting or “dry runs”** | *Population tested (naive content experts and/or sample of the target learner population), number of tests...* | | | | |
| **Any element that can improve the scenario following the simulation session** | *What are the elements that educators can change to improve the scenario (to be filled in after the simulation session)?* | | | | |

**APPENDIX 2. Simulated participant based immersive simulation template (translated from French)**

| 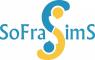  *Institutional logo*  **SIMULATED PARTICIPANT BASED IMMERSIVE**  **SCENARIO (SP)**  ***Scenario title (specify)*** | | | | |
| --- | --- | --- | --- | --- |
| *This scenario template is designed within the framework of an immersive simulation with a simulated participant (PS) and is centered on care relationships. This type of simulation involves a simulated participant (standardized or not) around whom the scenario is designed. Learners interact mainly with this simulated participant and sometimes other participants (family, colleagues, etc.)*. | | | | |
| **SCENARIO CONTEXT** | | | | |
| Simulation institution | *Name of the training institution* | | | |
| Author(s) | *Person(s) who designed the scenario* | | | |
| E-mail | *Contact person's e-mail for the scenario* | | | |
| Revision date | *Date of last scenario revision* | | | |
| Educational team (specify if trained in simulation) | *Technicians, facilitators, instructors, simulator voices...* | | | |
| Brief summary for TRAINERS | *Scenario description exclusively for use by the educational team* | | | |
| Target learners | Number | Function (profession, attire) | | Prior experience with simulation |
|  |  |  | |  |
| Possible link to a training program or curriculum | *Where does the scenario fit into the learners' training program? Professional development, initial training with a formal curriculum, postgraduate training...* | | | |
| Simulated participant(s) | *Number, specific training, name of association or school if any, etc.* | | | |
| **TRAINING OBJECTIVES** | | | | |
| Specific educational needs of the learners | | | | |
| *Specify the method and results of the needs analysis that led to this scenario: “What justifies this scenario?”* | | | | |
| **Learning objectives (3 to 5 max)** | | | | |
| Technical skills | | Non-technical skills | | |
| *Healthcare expertise adapted to the learners*  *Be able to ...* | | *Human factors adapted to the learners*  *Be able to...* | | |
| Possible emerging learning objectives | | | | |
| *Specify here any potential emerging objective that could be debriefed if necessary (to help prepare for debriefing).* | | | | |
| **SCENARIO PREPARATION** | | | | |
| Provided handouts during simulation, if needed | *Documents, patient chart, bio, ECG, imaging...* | | | |
| Learning aids to be provided to learners before and/or after the training session | *Cognitive aids or documentation from the available literature or developed by the educational team* | | | |
| Bibliographical references or recommendations related to the scenario | *Specify here, if available, the bibliographical references on which the scenario is based* | | | |
| Choice of the simulation environment | *In situ, emergency room, hospitalization room, intensive care unit, etc.* | | | |
| Description of the equipment required in the simulation room with a level of realism adapted to the learners’ expertise | *Specify equipment required in the simulation room* | | | |
| Prebriefing at the start of the training session | *Invitation modalities, prebriefing adapted to the learners' simulation experience, specific information about the training session in which the scenario is integrated (e.g. death in simulation, etc.), prebriefing checklist used (to adequately prepare learners and avoid difficult debriefings).* | | | |
| **SIMULATION PROCESS** | | | | |
| Simulation duration | *Approximate duration of the simulation session, especially to fit the schedule of the day if necessary* | | | |
| Briefing for the SIMULATED PARTICIPANT(S)  (detail only with relevant information for the scenario) | ***SP script***  *Information to be provided to the learners only upon request is indicated* ***in bold****.*  *Other information is provided spontaneously****.***  *Background/reason for the SP visit/complaint/request:*  *Type of SP: specify if necessary (male or female / age group)*  *Identity of the SP: (surname, first name, date of birth, age)*  *Personal history: (hospital, surgery, etc.)*  *Allergies:*  *Current treatment:*  *Family history:*  *Weight/height/BMI:*  *Other if required:*  *Marital status:*  *Occupation/leisure:*  *Consumption of tobacco/alcohol/drugs:*  *Last meal (time, content):*  *Pain/symptoms (if applicable):*  *The onset of symptoms, duration:*  *Intensity of symptoms:*  *Location, radiation of pain:*  *Aggravating/enhancing/precipitating factors:*  *What the SP thinks/believes/imagines:*  *Analgesic position/attitude towards pain:*  *SP status / behavior / communication skills:*  *Speech: (comatose, unable to speak, asleep, silent, calm, normal, out of breath, confused, incoherent, loud...)*  *Emotional state: detail what you expect from the SP in terms of verbal language, non-verbal language, body language, physical attitude, facial expression, eye contact or not.)*  *Expression of an emotion with its intensity (0 to 10)*  *Clinical signs of SP if necessary: (heart rate, breathing rate, temperature, blood pressure)*  *Data to be found on clinical examination* | | | |
| Attire of the SP(s)  +/- physical preparation (if necessary) and positioning on arrival of the learners | *Positioning: stretcher/bed/floor/wheelchair/sitting/standing/other: ..........*  *Make-up/wound/bandages:*  *Specify attire, if important for the scenario.* | | | |
| Briefing for LEARNERS | *Reason for consultation: context, information known about the SP (history and current medical history)*  *Any relevant information without divulging the scenario.* | | | |
| *The following section will help you identify all the behaviors you want to observe in your learners concerning the scenario objectives. It is then necessary to identify all the triggers for these behaviors and anticipate their consequences or impact on the evolution of the situation.*  ***Insert or delete as many lines as you need, based on your objectives*** | | | | |
| Preparatory phase for learners  (“immersion chamber”) | *Dedicated concentration time for learners before starting the simulation, preferably outside the simulation room and in a quiet environment* | | | |
| Starting sentence | *Phrase or sentence uttered by the PS showing the beginning of the simulation session* | | | |
| Status / Duration (D) /  Trigger Event (TE) | **Observable learner behavior** | | **Consequences on the situation (reaction of the SP, phrases/words in response, etc.)** | |
| State 1: *example: Initial care*  D: *5 minutes*  ED: *Arrival of the medical team* | *Example: presentation of learners positioning themselves in the room* | | *Planned responses*  *SP reaction to the learners’ behavior* | |
| State 2:  D:  TE: |  | |  | |
| State 3:  D:  TE: |  | |  | |
| State 4:  D:  TE: | *Provide as many TEs or states as necessary for the scenario* | |  | |
| *Method of ending and exiting the scenario* | | | | |
| Preparation for debriefing | *1- Approximate planned debriefing duration*  *2- Strategies for resolving the simulated situation (how to behave, react, adapt, etc.), emerging problems, etc.*  *3- Prepare strategies for transfer of learning to practice = plan some elements of recontextualization, i.e. show that the strategies used in the simulation are valid for other situations (including real-life situations), sometimes relying on the learners’ lived experiences if any, by analyzing with them what was identical and what was different from the simulated situation...* | | | |
| **QUALITY** | | | | |
| **History of scenario piloting or “dry runs”** | *Population tested (naive content experts and/or sample of the target learner population), number of tests...* | | | |
| **Any element that can improve the scenario following the simulation session** | *What are the elements that educators can change to improve the scenario (to be filled in after the simulation session)?* | | | |

**APPENDIX 3. Procedural simulation scenario (translated from French).**

| *Institutional logo*  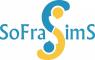  **PROCEDURAL SIMULATION SCENARIO**  ***Scenario title (specify)*** | | | |
| --- | --- | --- | --- |
| **PREAMBLE TO DOCUMENT DRAFTING** | | | |
| *1. Clearly state the LEARNING OBJECTIVES for each procedure.*  *2. Choose the SIMULATION MODEL: mannequin alone or hybrid simulation with a simulated patient (see simulated participant scenario).*  *3. VARY ACTIVITIES to diversify the task to complete, with the possibility of using Mastery Learning as a learning strategy (see section or learning strategies).*  *4. Maintain the AUTHENTICITY of the procedure to facilitate the transfer of learning to practice: use the same equipment as in the practice environment, when possible, maintain an environment close to the reality of the practice setting if possible, and ideally provide a brief scenario to contextualize the activity.*  *5. Teaching the PROCEDURE = giving feedback on the procedure as a whole, not merely on an individual action itself or its outcome (success/failure of the action).*  *6. Include an ASSESSMENT INSTRUMENT for the procedure*  *7. Anticipate strategies to prevent SKILL DECAY from the early stages:*  *- Allow SELF-REGULATED LEARNING: enable learners to rehearse procedures outside scheduled periods to promote deliberate practice leading to expertise (to be organized with the institution).*  *- Schedule MAINTENANCE SESSIONS for skills every 6 or 12 months, allowing learners to freely practice on the simulators of their choice to review different procedures.*  *- Teach MENTAL IMAGERY to be used before or after sessions to mentally rehearse a procedure.* | | | |
| **SCENARIO CONTEXT** | | | |
| Simulation institution | *Name of the training institution* | | |
| Author(s) | *Person(s) who designed the scenario* | | |
| E-mail | *Contact person's e-mail for the scenario* | | |
| Revision date | *Date of last scenario revision* | | |
| Educational team (specify if trained in simulation) | *Technicians, facilitators, instructors, simulator voices...* | | |
| Brief summary for TRAINERS | *Scenario description exclusively for use by the educational team, with a detailed description of the clinical context in which the procedural simulation activity will take place* | | |
| Target learners | Number | Function (profession, attire) | Required number of exposures before the training session |
|  |  |  |  |
| Possible link to a training program or curriculum | *Where does the scenario fit into the learners' training program? Professional development, initial training with a formal curriculum, postgraduate training...* | | |
| **TRAINING OBJECTIVES FOR THE TARGETED PROCEDURE** | | | |
| Specific educational needs of the learners | | | |
| *Specify the method and results of the needs analysis that led to this procedural training: "What justifies this training activity?”* | | | |
| **Learning objectives** | | | |
| *Specify the educational objectives for the procedure* | | | |
| **CONSIDER TEACHING STRATEGY(IES)** | | | |
| *1. Discovery learning (self-experimentation of the procedure with problem-solving)*  *2. Demonstration or exemplification (observation and imitation)*  *3. Mastery learning (learning in steps of increasing difficulty until mastery is achieved, with a valid assessment after each step)*  *4. Deliberate practice (autonomous practice with free access to the simulator once learning has been completed, with feedback and defined objectives)* | | | |
| **SCENARIO PREPARATION** | | | |
| Learning aids to be provided to learners before and/or after the training session | *Videos demonstration, mental imagery, cognitive aids, etc.* | | |
| Bibliographical references or recommendations related to the procedure | *Specify here, if available, the bibliographical references on which the procedure is based* | | |
| Choice of the simulation environment | *Type of room to be reproduced, layout, simulation location (in situ / at the sim center), etc.* | | |
| Description of the equipment required in the simulation room | *Type and number of dummies and consumables*  *Consider a hybrid simulation with an actor if relational objectives are added (communication, etc.).* | | |
| Simulator: learner ratio | *Number of learners per simulator* | | |
| Prebriefing at the start of the training session | *Invitation modalities, prebriefing adapted to the learners' simulation experience, specific information on the procedure to be learned, and prebriefing checklist used (to adequately prepare learners and avoid difficult debriefings).* | | |
| Preparing the simulator | *Presentation of the simulator (clothing, positioning, perfusion, consumables, etc.)* | | |
| Available paraclinical tests, if appropriate | *Anticipate imagery tests related to the clinical case, especially in digital format: X-rays, CT scans, MRIs, ultrasound, etc.* | | |
| **SIMULATION PROCESS** | | | |
| Detail and duration of the expected steps for the training session | *Example(s): video sequence, demonstration, performance, assessment, etc. (in line with the teaching strategy(ies))* | | |
| Learner briefing | *Case presentation, medical and surgical history, habitus, medication, morphological parameters, etc.*  *Define the roles of learners per simulator if necessary (guide, assessor, helper, co-participants at the same level, etc.).* | | |
| Assessment instrument | *To be designed or reused from literature: should contain*   - *Key elements of the teaching procedure* - *Determination of a minimum passing score* - *Bibliographical reference(s) for its design, if applicable* | | |
| Feedback | *Close supervision by one or more instructors (if so, how many instructors are needed?)*  *Self-learning (free access to simulators organized with the training institution)*  *Global feedback at the end of the activity (in groups, +/- video review)*  *Peer feedback* | | |
| **QUALITY** | | | |
| **Any element that can improve the scenario following the simulation session** | *What can the educators improve in the scenario (to be filled in at the end of the simulation session)?* | | |
| **Simulator quality control** | *Simulator use tracking sheet (potential problems, simulator damage, consumables used, etc.)* | | |
| **Strategies to prevent skill decline** | *New sessions to be scheduled, frequency, mental imagery, etc.* | | |
